# SIRT2 plays complex roles in neuroinflammation neuroimmunology-associated disorders

**DOI:** 10.3389/fimmu.2023.1174180

**Published:** 2023-05-05

**Authors:** Wenmei Lu, Haonan Ji, Danhong Wu

**Affiliations:** Department of Neurology, Shanghai Fifth People’s Hospital, Fudan University, Shanghai, China

**Keywords:** SIRT2, apoptosis, necroptosis, autophagy, neuroinflammation, neuroimmunology, cerebral ischemic stroke, neurodegenerative disease

## Abstract

Neuroinflammation and neuroimmunology-associated disorders, including ischemic stroke and neurodegenerative disease, commonly cause severe neurologic function deficits, including bradypragia, hemiplegia, aphasia, and cognitive impairment, and the pathological mechanism is not completely clear. SIRT2, an NAD^+^-dependent deacetylase predominantly localized in the cytoplasm, was proven to play an important and paradoxical role in regulating ischemic stroke and neurodegenerative disease. This review summarizes the comprehensive mechanism of the crucial pathological functions of SIRT2 in apoptosis, necroptosis, autophagy, neuroinflammation, and immune response. Elaborating on the mechanism by which SIRT2 participates in neuroinflammation and neuroimmunology-associated disorders is beneficial to discover novel effective drugs for diseases, varying from vascular disorders to neurodegenerative diseases.

## Introduction

Neuroinflammation and neuroimmunology-associated disorders involve cerebral vascular diseases, neurodegenerative diseases, neuroinflammatory diseases, intracranial infectious diseases, traumatic brain injury, etc. Cerebral ischemic stroke and neurodegenerative diseases are the most common neuropathological disorders, which bring heavy burden to society and families (1, 2). The silent information regulator 2 (SIRT2), a cytoplasmic NAD^+^-dependent deacetylase, plays important roles in both cerebral ischemic stroke and neurodegenerative diseases (3–5).

Increasing evidence has elucidated various pivotal roles that SIRT2 plays in pathological processes, including apoptosis, necroptosis, autophagy, and Inflammatory immune response which were the pathological mechanism underlying cerebral ischemic stroke and neurodegenerative diseases (6–8). And SIRT2 is highly expressed in the brain. It is mainly expressed in Myelin-Rich Regions in Oligodendrocytes in brain (9). SIRT2 protein also expresses in neurons even though its biological function is not completely clear (10). In addition, Werner et al. and Beirowski et al. found SIRT2 could regulate myelin formation in nervous system (11, 12). The higher SIRT2 expression in brain suggests that SIRT2 regulates the pathophysiologic progress in the central nervous system.

**Figure 1:**
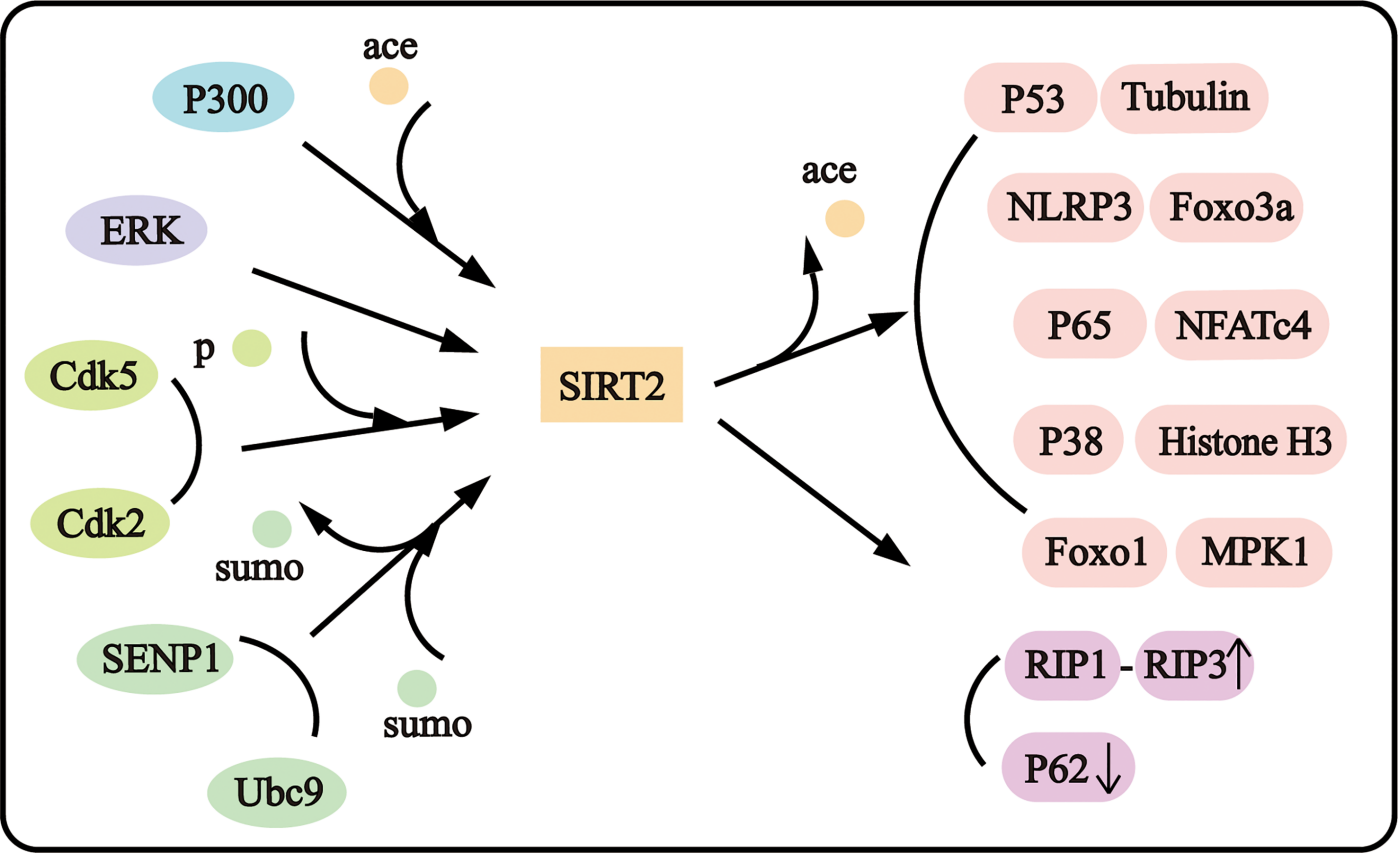
SIRT2 interacts with diverse proteins.

SIRT2, as an important deacetylase, was proved to colocalize with microtubules mainly in the cytoplasm. SIRT2 deacetylated tubulin at lysine 40 with preferential affinity than histone H3 (3). And Tubulin involves in various pathophysiological processes such as cytoskeletal maintenance, axonal degeneration, and axial transport (13, 14). P38 was proved to be one of SIRT2 substrates, SIRT2 deacetylated P38 to suppress neuroblastoma (15). SIRT2 could deacetylate Forkhead Box Protein O3a (Foxo3a), P53, Foxo1, Nuclear Factor Of Activated T Cells 4 (NFATc4), P65, and the NLR Family Pyrin Domain Containing 3 (NLRP3) to influence cell viability and inflammation. P300 is the most important acetylase in mammals, and SIRT2 could regulate P300 autoacetylation (16). In addition, SIRT2 could be regulated as the substrate. P300 could acetylate SIRT2 and attenuate its deacetylase activity (17), and the cyclin-dependent kinase 5 (Cdk5) could promote the phosphorylation of SIRT2 at Ser331 and 335 sites (18) and cyclin E-Cdk2, cyclin A-Cdk2 could phosphorylate SIRT2 at Ser331 to inhibit its catalytic activity (19). Mitogen-Activated Protein Kinase 3/1 (ERK1/2) and was reported to interact with SIRT2 and induce the activity, stability, and protein levels of SIRT2 (20). And SIRT2 could be sumoylated, and desumoylated-SIRT2 possesses lower deacetylase activity (15). The complex protein interaction with SIRT2 may constitute the complex regulatory network of SIRT2 in neuropathological disorders (**Figure 1**).

### SIRT2 in ischemic stroke

Acute ischemic stroke (AIS) commonly causes hemiplegia, aphasia, paresthesia, coma, and even death. It is important to discover the pathological mechanism of ischemic stroke. Recent studies have shown acute ischemic stroke promotes SIRT2 protein expression. In ischemic stroke mouse brains, expression of SIRT2 was upregulated and translocated into neuronal nuclei in the ischemic penumbra (21). But in photothrombotic stroke rats, expression of SIRT2 was upregulated in the cytoplasm of the penumbra neurons and SIRT2 was not detected in penumbra astrocytes (22). The serum SIRT2 expression was increased in AIS patients alongside increasing serum Tumor Necrosis Factor (TNF), IL-6, and IL-17. Serum SIRT2 expression was positively correlated with deteriorated neurological function (23). SIRT2 induces neuron death after ischemic stroke. AK1 and AGK2 could downregulate the AKT/FOXO3a pathway and reduce cleaved caspase-3, Bim, and Bad, resulting in attenuating neuron apoptosis induced by middle cerebral artery occlusion (MCAO) (5). Down-regulation of SIRT2 by inhibitor or SIRT2 knockout could decrease the infarct volume and neurological impairment scores (5, 21). Another SIRT2 inhibitor AK7 showed a neuroprotective effect by activating the P38 MAPK signaling pathway in MCAO mice (24). However, the SIRT2 inhibitor, SirReal2, did not change the infarction volume in photothrombotic stroke rats (22). Nicotinamide phosphoribosyltransferase (Nampt) is the rate-limiting enzyme for mammalian NAD synthesis. Nampt and NAD showed a protective effect in increased neural stem cells, lower mortality, improved neurofunctional deficit, and enhanced body weight after middle cerebral artery occlusion. And SIRT2 regulated the Nampt-NAD axis-dependent prodifferentiative effect (25).

Neuroinflammation and immune response contribute to the pathophysiology after ischemic stroke. Infiltrating regulatory T cells (Treg Cells) provide neuronal protection in the penumbra region. And the microglia induced the expression of SIRT2 and hypoxia-inducible factor 1-alpha (HIF-1α) in Treg Cells to weaken its anti-inflammatory effect after MCAO (26). However, SIRT2 could enhance NAD/NADH mediated ATP increases in microglia (27) and prevent excessive microglia activation-induced inflammation through NF-κB deacetylation (28). The inflammasome is a multi-protein complex to recruit and activates proinflammatory caspase-1, followed by the secretion of inflammatory cytokines and other mediators (29). The microglia-specific Microglia-specific PPAR gamma coactivator-1alpha (PGC-1α) could decreased neurologic deficits of acute ischemic stroke mice by inhibiting inflammatory response (30). Glycogen synthase kinase 3β (GSK-3β) inhibitor and knockdown improved neurological function and reduced infarction volume by reducing NLRP3 inflammasome, cleaved caspase-1, IL-1β, and IL-18 in MCAO rats (31). SIRT2 was proved to inhibit NLRP3 inflammasome depending on its deacetylate activity suggesting SIRT2 may influence ischemic stroke by regulating inflammasome activity.

### SIRT2 in neurodegenerative disease

The neurodegenerative disease involves a range of chronic and progressive disorders that are characterized by misfolding proteins deposition, loss, and dysfunction of neurons, neuroinflammation and immune homeostasis destruction (32). Parkinson’s disease is one of the most common Neurodegenerative disorders with distinctive manifestations of static tremor, rigidity, and bradykinesia (33). The serum SIRT2 expression was positively correlated to Parkinson’s disease, and the serum SIRT2 level could help to discriminate Parkinson’s disease from atypical Parkinson’s syndrome (34). YKK(ε-thioAc)AM, one of the SIRT2 inhibitors, could inhibit the serum SIRT2 of Parkinson’s disease patients (35). SIRT2 Inhibition has been considered a neuroprotective effect in Parkinson’s disease. Lei L et al. demonstrated SIRT2 exacerbates neuronal apoptosis and nigrostriatal damage by deacetylating Foxo3a and activating Bim both *in vitro* (36) and *in vivo* Parkinson’s disease (PD) model (37). SIRT2 level was highly increased in the PD model, and miR-212-5p could inhibit SIRT2-P53 axis-dependent programmed cell death in PD pathogenesis (38). In addition, SIRT2 was observed to translocate to the nucleus in both cellular and animal PD models. The cyclin-dependent kinase 5 (Cdk5) phosphorylated SIRT2 at the Ser331 and Ser335 sites and promoted SIRT2 nuclear translocation, and subsequently caused neuronal death and PD progression (18). SIRT2 inhibition could rescue alpha-synuclein toxicity and protect against dopaminergic cell death in the Drosophila PD model (39). The ICL-SIRT078, a selective SIRT2 inhibitor, showed neuroprotective function *in vitro* PD model (40). And AK7, another SIRT2 inhibitor, ameliorated alpha-synuclein toxicity and reduced dopaminergic neuron loss in Parkinson’s disease (41). But there have been few studies that proposed different conclusions. Éva M Szegő et al. found that SIRT2 interacted with protein kinase B and modulated DA neurons differentiation. SIRT2 knockout decreased the number of DA neurons in the substantia nigra and reduced striatal fiber density in mice (42). And Preeti Singh et al. found SIRT2 could reduce the formation of α-synuclein and enhance SOD2 expression to prevent neuronal stress in the PD brain (43). These results suggest that SIRT2 may mediate the pathogenesis of Parkinson’s disease with intricacy and important mechanisms.

SIRT2 plays important role in other neurodegenerative diseases. AK7 remarkably reduced aggregated mutant huntingtin, improved motor function, reduced brain atrophy, and extended survival of genetic mouse models of Huntington’s disease (44). And a thiazole-containing inhibitor of SIRT2 induced Nuclear respiratory factors 2 (NRF2) activation and reduced production of reactive oxygen species and nitrogen intermediated in Huntington’s disease (45). However, another study showed SIRT2 deletion in the gene could not influence mouse Huntington’s progression in the mouse model (46). It was found the level of SIRT2 was increased in the plasma of Alzheimer’s disease (AD) patients than control subjects (47). And SIRT2 level was found positively related with p-tau level in the cerebral fluid of AD patients (48). The risk of Alzheimer’s disease has been reported to be correlated with the single-nucleotide polymorphism (SNP) at the 3’un-translated region (3’UTR) of SIRT2 (49). And SIRT2 inhibition exhibited neuroprotection in Alzheimer’s disease. And SIRT2 was enhanced in insulin-deficient amyloid-β (Aβ) precursor protein (APP) transgenic mouse model, and overexpression of SIRT2 induced tau hyperphosphorylation through ERK activation (50). The SIRT2 inhibitor, AK1 or SIRT2 knockout could increase the elimination of Aβ oligomers to enhance cell survival by increasing microtubule stabilization and improving autophagy (51). Ning B et al. found that SIRT2 inhibition could enhance the acetylation of the amyloid precursor protein, increase soluble APP-α (sAPPα) protein, and inhibit β-amyloid-induced neuron toxicity to ameliorate cognitive impairment (52, 53). Based on the above reports, most studies showed SIRT2 inhibition plays a neuroprotective effect in neurodegenerative disease.

## SIRT2 in apoptosis

Apoptosis is the most fundamental form of cell death which is called programmed cell death possessing special morphological features as their characteristic. The characteristic morphology changing of apoptosis includes cytoplasmic shrinking, activation of the caspase-3 pathway, fragmentation of chromosomes, nuclear membrane breakdown, and formation of apoptotic bodies, and consequently the cell breaks up (54). Importantly, apoptosis has been widely observed in neuropathological disorders. A large amount of reactive oxygen species (ROS), DNA damage, mitochondrial dysfunction, and Ca^2+^ overload caused by neuropathological progression could induce cell apoptosis (55, 56).

MPTP (1-methyl-4-phenyl-1,2,3,6-tetrahydropyridine) is a dopaminergic neurotoxin causing nigrostriatal damage which is detected to be located in the central nervous system (CNS) after system administration. Knockout of SIRT2 could lead to the increase of Foxo3a acetylation and the decrease of tyrosine-hydroxylase and Bim expression levels, reduce of MPTP-induced apoptosis (36). Yongzhi L et al. reported noise exposure upregulated cochlea SIRT2 expression and SIRT2 inhibition attenuated the noise-induced hearing loss (NIHL). The AK7, a selective SIRT2 inhibitor, reduced oxidative nuclear DNA damage and cochlear cells apoptosis (57). Hui N and her colleagues found that down-regulation of SIRT2 could significantly alleviate cells early-stage apoptosis induced by H_2_O_2_ in PC12 cells. They detected that siRNA and AGK2 decreased apoptosis by attenuating H_2_O_2_-inducing caspase-3 activation and a decrease in ROS levels resulting from H_2_O_2_ application (58). Melatonin might suppress SIRT2-mediated FOXO3a deacetylation, reduce pro-apoptotic proteins, and increase Bcl-2 and Bcl-2/Bax in the hippocampus of aged rats (59). Above all, SIRT2 induces cell apoptosis in a pathological state.

Oppositely, other studies showed that SIRT2 inhibition may exacerbate cell apoptosis in a given pathological progression. Using Sirtinol and Salermide to inhibit SIRT2, P53 acetylating was increased as well as cell apoptosis was enhanced (60). AEM1 and AEM2, two selective SIRT2 inhibitors, increased p53 acetylation levels and up-regulated CDKNIA (Cyclin-dependent kinase inhibitor 1A gene) expression, and then CDKNIA targets with the cell cycle regulation element p21WAF11 and two pro-apoptotic genes PUMA and NOXA to mediate apoptosis (61). Furthermore, it is proved that Sirt2 deacetylate lysine residues in the catalytic domain of P300 indirectly influence p53 activity (16). Additionally, Sirt2 deacetylated and down-regulated the transcriptional activity of P53, and 14-3-3 β/γ augment deacetylation and down-regulation of P53 transcriptional activity by Sirt2 in an AKT-dependent manner (62). Further study showed that SIRT2 catalyzed P53 deacetylation in the cytoplasm at lysine 382 (63). And SIRT2 inhibition showed an induced-apoptosis effect in the vascular endothelial cell line (PIEC). Jie Z et al. showed both SIRT2 inhibitor and SIRT2 siRNA could induce mitochondrial depolarization and decrease intracellular ATP levels (64).

## SIRT2 in necroptosis

Necrosis is initially known as a passive energy-independent cell death pathway that is inversely proved that at least partial forms of necrosis are well-regulated. There is evidence existing to elucidate the molecular regulation mechanism in the specific caspase-independent programmed necrosis. TNF-inducing necroptosis can activate receptor-interacting protein 3 (RIP3), and activating RIP3 further promotes receptor-interacting protein 1 (RIP1) phosphorylation, subsequently, RIP1-RIP3 complex formation. The complex can activate the pro-necrotic kinase, promote reactive oxygen species production, and finally, programmed necrosis occurring (65). Necroptosis is important in responding to environmental insults, for example, viruses or bacteria infection, and acute ischemia stroke (66). And current studies suggest that necroptosis is associated with neuroinflammatory disease (67). As for neurodegenerative disease, applying methamphetamine to embryonic cortical neurons, significant necroptosis was found in a time and dose-dependent manner with over-expression of tumor necrosis factor-α (68).

Knockdown of SIRT2 could reduce the RIP1-RIP3 complex formation in response to TNF inducing programmed necrosis in a casepase-8 activity manner (69). And SIRT2 down-regulation was proved to attenuate necroptosis by inducing the acetylation and nuclear translocation of the nuclear factor of activated T-cells 4 (NFATc4) (70), the acetylation of mitogen-activated protein kinase phosphatase-1 (MKP-1) and suppressing the phosphorylation of p38 and JNK (71). Confusingly, knockdown of SIRT2 by AGK2 or three independent siRNAs failed to prevent cells from necroptosis in L929 cells, mouse embryonic fibroblasts (MEFs), and bone marrow-derived macrophages (BMDMs) induced separately by TZ and TNF (72). So more works are warranted to elucidate the roles and underlying mechanisms of SIRT2 in necroptosis.

### SIRT2 in autophagy

Autophagy is a self-digestion process of denatured proteins and damaged organelles. And autophagosomes with double membrane structures are gradually informed and delivered to lysosomes (73). Autophagy involves various complex autophagy protein network pathway that influences autophagosome formation and maturation (74). Appropriate autophagy plays neuroprotective effects on cerebral ischemia, while excessive autophagy deteriorates neurological injury (29). Autophagy activation help the elimination of the intracellular abnormal protein deposits, such as lewy bodies and α-Synuclein (75). So autophagy may be the potential target to regulate neurodegenerative and ischemic diseases.

Recent studies report that SIRT2 could deacetylate FOXO1. And the interaction between acetylated FoxO1 and Autophagy Related 7 (Atg7) is essential for autophagy induced by oxidative stress or serum starvation (76). Another paper elaborated that FoxO1 translocation through the cytoplasm and nuclear modulates time-dependent autophagy. It is noted that FoxO1 hypo-acetylation in nuclear and hyper-acetylation in the cytoplasm may induce autophagy. And SIRT2 could deacetylate FOXO1 in the cytoplasm to mediate autophagy (77). Jiyeong Gal et al. found that over-expression of SIRT2 inhibited lysosome-mediated autophagy by regulating autophagosome formation. And then, SIRT2 over-expression exacerbates MG132 and amyloid-inducing protein-mediated cytotoxicity, resulting in increased cell fragility in response to environmental stress. They also found SIRT2 down-regulation could reduce the level of ubiquitinated protein as well as cytotoxicity induced by MG132 in the siRNA-transfection-inducing SIRT2 silencing SH-SY5Y cell model (78). Consistently, a study reported that knockdown of autophagy genes (ATG5–ATG7) increased p62 accumulation and suppressed autophagy, and SIRT2 knockdown could attenuate p62 reduction so that basal autophagy levels were up-regulated in cells. SIRT2 knockdown would prevent post-slippage death, followed by mitotic arrest influenced by rapamycin and mild starvation which mediates autophagy up-regulation (79). These results suggested SIRT2 may suppress stress and starvation-induced autophagy.

Microtubule Associated Protein 1 Light Chain 3 (LC3) is the key marker of autophagy. The removal of the C-terminal 22 amino acids of cytosolic LC3-I to synthesize membraned bound LC3-II means autophagosome maturation. Song, T et al. found acetylation of LC3 highly influences its stability and cargo recognition ability. Acetylation inhibited the interaction of LC3 and P62 and the LC3 proteasome-dependent degradation process. In a word, acetylated LC3 is suitable for storage (80). The histone deacetylase 6 (HDAC6) could deacetylate LC3. And the HDAC6 inhibitor, tubacin could upregulate acetylated LC3 along with p62/SQSTM1 accumulation to reduce serum starvation-induced autophagy degradation (81). Moreover, there is a paper showing that SIRT1 could deacetylate nuclear LC3 at K49 and K51 to stimulate starvation-induce autophagy. The deacetylated LC3 could interact with the nuclear protein DOR and shuttle to the cytoplasm where it can bind Atg7 (82). As shown above, HDAC6 was not the only deacetylase acting on LC3B-II (81), and SIRT1 mainly deacetylated nuclear LC3. Consequently, there may be other deacetylases catalyzing LC3 in the cytoplasm, and SIRT2 is a deacetylate kinase mainly distributed in the cytoplasm. Whether SIRT2 involves in autophagy by promoting the deacetylation of cytoplasm LC3 remains unclear.

### SIRT2 in neuroinflammation

Inflammation is a double-edged sword. it protects organisms against viruses and bacteria invasion. But in other conditions, inflammation may exacerbate tissue injury, especially in the process of Systemic inflammatory response syndrome (SIRS). The role of SIRT2 on inflammation is still controversial and seems to be tissue-specific and context-dependent. Several groups reported that SIRT2 can inhibit inflammation in different pathological processes (28, 83, 84). SIRT2 deacetylates P65 at Lys310 (85). In LPS-stimulated inflammation, it has been found that SIRT2 knockout mice showed increases in microglia activation and pro-inflammation cytokines expression by enhancing acetylated NF-kB and reducing its phosphorylation at serine 331 (S331) (28). Han, B et al. found that PGC-1α could reduce the NLRP3 activation and proinflammatory cytokine production after acute ischemic stroke (30). Liu, M et al. found SIRT2 impairment activated the NF-κB signal pathway by deacetylation and phosphorylation of P65. Consistently, Another study reported SIRT2 inhibitor AK7 increased NF-κB P65 acetylation, resulting in increasing of aquaporin4 (AQP4), matrix metalloproteinases (MMP)-9, proinflammatory cytokines, and chemokines, which exacerbates neuroinflammation (86). And SIRT2 was proved to inhibit the NLRP3 inflammasome activation by deacetylating NLRP3. And deacetylation of NLRP3 by SIRT2 could inhibit aging-associated inflammation (87).

Other studies found the opposite results: Inhibition of SIRT2 can attenuate inflammation in macrophages (88) and microglia (89). SIRT2 inhibitor may inhibit the phosphorylation of IκBα, which can inhibit inflammation by decreasing the NF-κB activity (88, 90). Besides, SIRT2 inhibitors might also decrease inflammation by suppressing the MAPK signaling (91). Moreover, the SIRT2 inhibitor exhibited a protective effect for SH-SY5Y cells by inhibiting LPS-stimulated production of TNF-αand PGE2 from microglial cells (92). AGK2, a SIRT2 inhibitor, could decrease the LPS-induced increase of the mRNA of TNF-α and IL-6, the level of active Caspase-3 & Bax and iNOS levels, block NF-κB nuclear translocation, increase the expression of MKP-1 and inhibit the activation of BV2 microglia (93, 94).

There are also several studies that reported that SIRT2 could not affect inflammation. Cambinol, a nonselective inhibitor of Sirt1 and SIRT2, could inhibit the expression of cytokines (including TNF, IL-1β, IL-6, IL-12and IFN-γ), NO and CD40 induced by toxic shock syndrome in macrophages, DCs, splenocytes, and whole blood. However, cambinol and sirtinol may not inhibit inflammation by acting with just SIRT1 and SIRT2 because selective SIRT1 (EX-527 and CHIC-35) and SIRT2 (AGK2 and AK-7) inhibitors could not inhibit inflammation (95). By comparing wild and SIRT2 knockout C57BL/6 mice, no significant alteration in inflammatory cells activating was shown in the ischemic brain hemispheres between wild-type mice and SIRT2(-/-) genotype mice (9). It is also reported that autophagy could inhibit inflammasome activation and inflammatory responses through mTOR and AMPK pathways after ischemic stress (29). So SIRT2 may partly regulate inflammation by inhibiting autophagy.

### SIRT2 in neuroimmunology

The central nervous system had been viewed as an immune-privileged site (96). However, decades of studies have shown that the central nervous system is a rigorous immune regulatory tissue with both innate and adaptive immune cells. Neuro-immune response widely involves in both the development of the nervous system and neuropathological changes (97, 98). The central nervous system is protected by complex immune defense barriers constituted by the skull, meninges, blood-brain barrier (BBB), and cerebrospinal fluid (CSF) barrier (99). The skull contains special bone marrow niches connected to meningeal veins and dural sinuses which maintain the balance of immune cells and promote the rapid immune response to injury in the meninges (100). And the bone marrow pockets possess the tolerance to CNS antigens and can sign distinct developing cells (100, 101). The meninges contain various immune cells, including dendritic cells (DCs), natural killer cells, T and B cells, mast cells, innate lymphoid cells, neutrophils, and monocytes, and the immune cells can recruit from the meninges to the parenchyma in immune response (102, 103). The blood-brain barrier consists of various structures and cells, including vascular endothelial cells, basement membrane, astrocytic end-feet, and microglia (104). The BBB barrier limits pathogenic factors and the immune cells to reach the brain parenchyma from blood vessels (105). The choroid plexus is the main producer of cerebrospinal fluid. And the choroid plexus contains resident immune cells so that it could also regulate immune response (106). And the cerebrospinal fluid is important in parenchymal waste and antigen drainage, and immune surveillance (107, 108). Microglia are the main resident immune cells in the brain and the monocytes could be recruited quickly during the immune response and differentiate into DCs or macrophages (109, 110). Immune homeostasis is important in the physiological function maintenance of the central nervous system, and neuro-immunological response in the pathological processes involves multiple central nervous system diseases. For example, the amyloid-βinduces brain endothelial cell impairment and blood-brain barrier dysfunction in Alzheimer’s disease (111). And microglial activation, infiltration of immune cells, and deficit of blood-brain barrier integrity play vital roles in the damage of ischemic stroke (112).

Present studies have proved that SIRT2 plays an important role in neuroimmunology. SIRT2 directly mediates the AKT phosphorylation and intracellular ATP increasing in NAD-induced BV2 microglia (27). Downregulation of the SIRT2 level could inhibit the activation of microglia (93). And SIRT2 knockout help to maintain the integrity of BBB by inducing the expression of ZO-1 and reducing ZO-1 gaps (113). But another study showed that SIRT2 inhibitor could enhance the acetylation and nuclear translocation of NF-κB and upregulate AQP4 and MMP-9, followed by blood-brain barrier disruption (86). Recently reported, regulation T cells (Treg cells) infiltrated the brain 1 to 5 weeks after ischemic stroke in mice. Treg cells provided a neuroprotective effect by increasing microglial reparative activity, followed by oligodendrogenesis and white matter repair after ischemic stroke (114). And another study found SIRT2 expression was upregulated in Treg cells three days after transient middle cerebral artery occlusion. Inhibition of SIRT2 activity could upregulate the expression of immunosuppression-associated molecules and enhance the anti-inflammatory effect of Treg cells. Furthermore, microglia could induce the expression of hypoxia-inducible factor 1-alpha (HIF-1α) to enhance SIRT2 expression in Treg cells (26). And SIRT2 also regulates the immune response in neurodegenerative diseases. Sa de Almeida J et al. found that microglial SIRT2 protected the hippocampal by inhibiting N-methyl-D-aspartate (NMDA)-mediated excitotoxicity and affecting synaptic plasticity (115).

## SIRT2 inhibitors

With the deeper study of SIRT2 biological function, various specific inhibitors have been developed (**Table 1**). AK-1, a small molecule SIRT2 inhibitor, shows neuroprotective effects in a mouse model of frontotemporal dementia (FTD) by regulating the expression of mutant tau protein (116). AK-7 is the developed sulfobenzoic acid derivation analog of AK-1. It is a specific SIRT2 inhibitor that is permeable to the blood-brain barrier (117). AK-7 can competitively inhibit SIRT2 in NAD^+^ binding sites (41). AGK2 is an effective and selective SIRT2 inhibitor with an IC50 of 3.5μM. Its inhibition activity level remained weak at 10 times higher concentrations for SIRT1 or SIRT3. AGK2 showed neuroprotective function in ischemic stroke (5). AEM1 and AEM2 show an SIRT2 inhibition effect with IC50 values of 18.5 and 3.8μM, but both the two compounds showed weak inhibition activity to SIRT1, SIRT3, and yeast Sir2 (61). A recent study showed that Thiomyristoyl (TM), a SIRT2 selective inhibitor, exerts anticancer activity by degradation of c-Myc oncoprotein (118). YKK (ε-thioAc) AM is a designed pentapeptide inhibitor containing N-thioacetyl-lysine against SIRT2. YKK (ε-thioAc) AM was more specific towards SIRT2 than SIRT1 with the IC50 of 0.15μM (35). The 3-((2-methoxynaphthalen-1-yl)methyl)-7-((pyridin-3-ylmethyl)amino)-5,6,7,8-tetrahydrobenzo[4,5]thieno[2,3-d]pyrimidin-4(3H)-one(ICL-SIRT078) is a SIRT2 specific inhibitor with a Ki value of 0.62 ± 0.15 μM. ICL-SIRT078 inhibits SIRT2 in a substrate-competitive manner, and its affinity with SIRT2 was more than 50-fold selectivity against SIRT1, SIRT3, and SIRT5 (40). JH-T4 could inhibit SIRT2 by the formation of hydrogen bonding. Regretfully, JH-T4 could also inhibit SIRT1 and SIRT3 (119). These compounds inhibit SIRT2 with different mechanisms, which provides convenience for studying the pathophysiological mechanism of SIRT2.

**Table 1:** The enzyme activity of novel SIRT2 inhibitors.

Compound	Enzyme activity	Characteristic function
AK-1	IC50 = 12.5μM	
AK-7	IC50 = 15.5μM	Blood-brain barrier permeability
AGK2	IC50 = 3.5μM	AGK2 inhibits SIRT1 and SIRT3 with IC50s of 30 and 91 μM, respectively
AEM1	IC50 = 18.5μM	p53-dependently induces apoptosis
AEM2	IC50 = 3.8μM	
TM	IC50 = 28nM	TM inhibits SIRT1 with IC50 of 98 μM
YKK (ϵ-thioAc) AM	IC50 = 0.15μM	
ICL-SIRT078	Ki = 0.62 ± 0.15μM	substrate-competitive inhibitor
JH-T4	IC50 = 0.03 ± 0.01μM	JH-T4 inhibits SIRT2 by the formation of hydrogen bonding

## Conclusion and discussion

**Figure 2:**
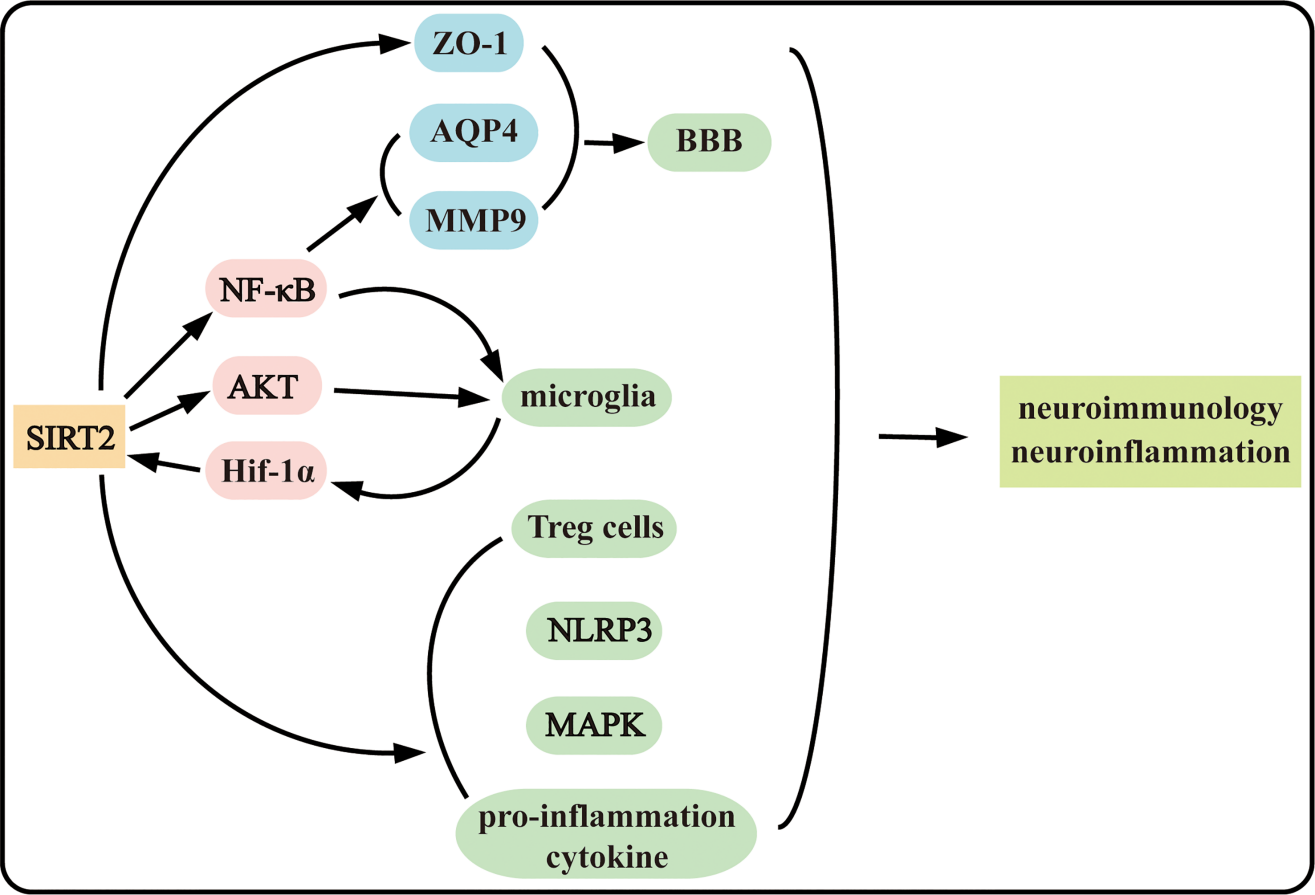
SIRT2 plays complex roles in neuroinflammation and neuroimmunology.

SIRT2 is playing an increasing role in cell death, including programmed cell death and non-programmed cell death, and neuroinflammation under complex mechanism (**Figures 1**, **2**). The functions of SIRT2 in necroptosis and neuroinflammation are still contradictory and more investigations are needed in the future to determine the roles and mechanisms of SIRT2. Moreover, SIRT2 plays intricate complex roles in ischemic stroke and neurodegenerative disease. Why does SIRT2 play a completely contradictory effect? Does SIRT2 play more roles in both biological maintaining and disorder occurrence? Further research is still necessary to uncover the mechanism SIRT2 acts underlying disease occurrence. And clarify the mechanism of SIRT2 in the pathogenesis is important in supplying novel potential therapy methods in the clinic. Discovering the mechanism that SIRT2 acts in disease could provide a new method to explore new drugs.

## Author contributions

DW helps to conceive and design the review. WL writes the paper. HJ helps to check and revise the paper. All authors contributed to the article and approved the submitted version.
